# A recent update on development, synthesis methods, properties and application of natural products derived carbon dots

**DOI:** 10.1007/s13659-023-00415-x

**Published:** 2023-11-13

**Authors:** Soumitra Sahana, Anupam Gautam, Rajveer Singh, Shivani Chandel

**Affiliations:** 1 Department of Pharmacognosy, ISF College of Pharmacy, Ghal-Kalan, Moga, Punjab 142001 India; 2 Institute for Bioinformatics and Medical Informatics, University of Tübingen, Sand 14, 72076 Tübingen, Germany; 3 International Max Planck Research School “From Molecules to Organisms”, Max Planck Institute for Biology Tübingen, Max-Planck-Ring 5, 72076 Tübingen, Germany; 4 Cluster of Excellence: EXC 2124: Controlling Microbes to Fight Infection, University of Tübingen, Tübingen, Germany

**Keywords:** Natural carbon dots (NCDs), Photoinduced electron transfer, Aggregation-Induced-Emission (AIE), Cancer therapy, Fluorescence, Bio-imaging, Sensing, Drug delivery

## Abstract

Natural resources are practically infinitely abundant in nature, which stimulates scientists to create new materials with inventive uses and minimal environmental impact. Due to the various benefits of natural carbon dots (NCDs) from them has received a lot of attention recently. Natural products-derived carbon dots have recently emerged as a highly promising class of nanomaterials, showcasing exceptional properties and eco-friendly nature, which make them appealing for diverse applications in various fields such as biomedical, environmental sensing and monitoring, energy storage and conversion, optoelectronics and photonics, agriculture, quantum computing, nanomedicine and cancer therapy. Characterization techniques such as Photoinduced electron transfer, Aggregation-Induced-Emission (AIE), Absorbance, Fluorescence in UV–Vis and NIR Regions play crucial roles in understanding the structural and optical properties of Carbon dots (CDs). The exceptional photoluminescence properties exhibited by CDs derived from natural products have paved the way for applications in tissue engineering, cancer treatment, bioimaging, sensing, drug delivery, photocatalysis, and promising remarkable advancements in these fields. In this review, we summarized the various synthesis methods, physical and optical properties, applications, challenges, future prospects of natural products-derived carbon dots etc. In this expanding sector, the difficulties and prospects for NCD-based materials research will also be explored.

## Introduction

Fluorescent nanoparticles, denoted as carbon dots (CDs), came into initial revelation in the year 2004, emerging from the process of refining single-walled carbon nanotubes via preparative electrophoresis. However, significant attention from researchers wasn’t garnered until the term carbon quantum dots was coined in 2006. The label carbon dots are specifically employed to differentiate these nanoparticles from the more extensive category of carbon nanoparticles, including carbon black [1, 2]. Myriad carbon allotropes have commanded considerable focus owing to their extensive potential utilities encompassing electronic apparatuses, biosensing tools, and agents for bioimaging. Various forms of graphitic architectural materials, including one-dimensional cylindrical carbon nanotubes (CNTs), diamond nanocrystals (DNs), zero-dimensional spherical fullerenes, as well as carbon dots (CDs) and two-dimensional graphene quantum dots (GQDs) and graphene, have been extensively studied over the past decade [3]. Carbon dots are a recently discovered type of fluorescent carbon material, characterized by their small size with a diameter below 10 nm. They are emerging as a potential substitute for metal-based quantum dots due to their unique composition and biocompatibility. CDs have gained attention for their applications as biosensors, gene carriers, drug transporters, and bioimaging agents, primarily because of their remarkable fluorescence properties, excellent biocompatibility, and low toxicity [4, 5]. The inaugural identification of carbon dots (CDs) transpired amid the refinement procedure of singular-walled carbon nanotubes engendered via methodologies involving arc discharge. Over the past few years, researchers have developed a range of starting materials and synthetic techniques to obtain CDs. These methods include electrochemical synthesis, supported routes, combustion or heating processes, hydrothermal approaches, acidic oxidation, microwave or ultrasonic treatments, arc discharge, laser ablation, and plasma treatment [3]. In 2010, the synthesis and purification of highly crystalline CDs were achieved, revealing size-dependent photoluminescence properties. This development marked an important milestone in the field. In recent years, researchers have been focusing on producing CDs with well-defined chemical structures and controlled morphologies. Some notable examples include crystalline C3N CDs, chiral CDs, triangular CDs and polymer-carbonized dots. These advancements in the synthesis and engineering of CDs open up new possibilities for tailoring their properties and expanding their applications in various fields [5, 6]. CDs, typically regarded as zero-dimensional nanostructures of carbon, generally exhibit a size smaller than 20 nm. They are composed of a combination of sp^2^/sp^3^ carbon atoms, forming a carbon skeleton, along with an abundance of functional groups and polymer chains. The assemblage of surface groups and polymer chains, encompassing entities like carboxyl, hydroxyl, and amine, synergistically bestows upon CDs exceptional aqueous solubility and facilitates seamless amalgamation with alternative substances devoid of partitioning. Additionally, the profusion of functional groups affords a facile avenue for the direct alteration of CDs with a myriad of organic or polymeric entities, thereby endowing them with adaptability across an extensive spectrum of sensing applications. Within the core framework of carbon dots (CDs), the central carbon configuration encompasses a composite of sp^2^ and sp^3^ carbon atoms, yielding either a lattice akin to graphite or adopting an amorphous carbon manifestation. This divergence arises from the disparate extents of carbonization that transpire during the CDs’ synthetic process. The covalent carbon skeletal structure intrinsic to CDs augments their stability, a pivotal facet for pragmatic utilization. This structural stability ensures that CDs can withstand various conditions and environments, making them reliable and suitable for a variety of uses [5, 7]. CDs have a significant advantage due to their easy and efficient synthesis process, which can be conducted on a large scale using a variety of carbon-containing precursor materials [8, 9]. These materials can be broadly classified into organic and inorganic carbon sources. While CDs synthesized from inorganic sources tend to have relatively low fluorescence quantum yields (QYs), additional surface passivation is often required. Therefore, organic synthetic precursors like ascorbic acid, malic acid, urea, citric acid, glucose and sodium citrate are commonly used to synthesize CDs [10–13]. The utilization of these precursors results in CDs exhibiting elevated QYs, coupled with the capability of incorporating diverse functional moieties, thereby augmenting luminescence. Nevertheless, the emphasis on the advancement of an environmentally conscious synthesis route for CDs centers on the adoption of natural protocols, sustainable solvents, and eco-compatible precursors encompassing organic natural constituents, biomass, and discarded matter. In contradistinction to an assortment of alternative fluorescent substances, CDs synthesized via green chemistry methodologies utilizing economical carbon reservoirs are profuse and ecologically benign [14]. Natural materials that are abundant in nature, easily accessible, continuously available, cost-effective, and renewable have the potential to replace commonly used chemicals in the synthesis of CDs. These materials offer numerous advantages, including their widespread distribution, easy availability, sustainability, affordability, and renewability. By harnessing these natural resources, the process of CDs synthesis can become more environmentally friendly and sustainable [15]. A wide range of precursors, including small molecules, natural polymers, and synthetic polymers, can be employed to form fluorescent CDs. These CDs can be synthesized through top-down or bottom-up methods. One-pot and multi-step approaches can be utilized to produce CD/photocatalyst composites. The recent progress in CDs has primarily centered around their applications in photocatalysis, particularly in pollutant degradation and energy-related areas [16].

## Synthesis methods for natural products derived carbon dots

These methodologies can be classified into two trajectories: the top-down and the bottom-up routes. The top-down avenue entails the disintegration of more extensive carbon constructs through physical or chemical methodologies. On the other hand, the bottom-up approach focuses on transforming smaller carbon structures into CD nanoparticles through chemical reactions. Various methods have been employed to prepare plant part-derived CDs, including hydrothermal synthesis, chemical oxidation, microwave synthesis, pyrolysis, solvothermal synthesis, and microwave-assisted synthesis (Fig. 3). These methods have utilized different plant parts such as leaves, fruits, flowers stems, roots, and seeds as starting materials for CD synthesis (Table 1). Among these methods, the hydrothermal method has gained significant popularity for synthesizing plant part-derived CDs. This technique involves subjecting the plant material to high temperature and pressure in an aqueous solution, resulting in the formation of CDs with unique properties. Each synthesis method offers distinct features and advantages. Chemical oxidation allows for the controlled production of CDs by selectively oxidizing the carbon precursor. Pyrolysis involves the thermal decomposition of organic matter to obtain carbon-based materials; including CDs. Hydrothermal synthesis provides a simple and efficient route for CDs formation, utilizing water as a reaction medium. Solvothermal synthesis employs organic solvents under high temperature and pressure conditions to produce CDs. Microwave synthesis and microwave-assisted methods utilize microwave irradiation to accelerate the reaction kinetics, enabling faster CD synthesis [17].

**Table 1:** Natural carbon dots from plant parts

Carbon dots from leaves				
Plant name	Scientific name	Used	Methodology	References
Spinach	*Spinacia oleracea*	Widely used in the analysis of blood, cell, microRNA, and environmental pollutants	Solvothermal Method	[92]
Basil	*Ocimum basilicum*	Exquisite applications	Pyrolysis Method	[93]
Green Tea	*Camellia sinensis*	Fluorescent sensor for gefitinib determination, Fluorescence sensing of gefitinib	Chemical oxidation, Pyrolysis Method, Microwave-Assisted Method	[61, 94]
Papaya	*Carica Papaya*	Carbon dots (CDs) demonstrated commendable water solubility, emitted blue fluorescence upon exposure to UV light, displayed excitation-dependent emission behavior, and possessed a nano-sized structure	Sand bath Method	[95]
Mulberry	*Morus*	Cultivating Silkworms for the Production of Highly Luminescent Silk	Hydrothermal Method	[96]
Cannabis	*Cannabis sativa*	The synthesized Ag@Carbon Dots demonstrated antimicrobial responsiveness in the presence of S. aureus and E. coli	Pyrolysis Method	[97]
Salvia miltiorrhiza	*Salvia miltiorrhiza*	Reactive Oxygen Species Scavengers for Mitigating Oxidative Plant Damage	Hydrothermal Method	[98]
Celery	*Apium graveolens*	Fluorescent paper sensors for the detection of nitrophenols, Sensors and bioimaging	Hydrothermal Method	[99]
kalmegh	*Andrographis paniculata*	Cellular imaging of human breast carcinoma cells (MCF-7)	Hydrothermal Method	[100]
Chinese mugwort	*Artemisia argyi*	Selective inactivation of Gram-negative bacteria	Smoking Simulation Method	[101]
Willow	*Salix spp.*	Fe3 + Detection	Hydrothermal Method	[102]
Mint	*Mentha*	Dual Analyte Recognition of Fe3 + and AA through a Selective and Sensitive Fluorescent Sensor with Detection Capability	Hydrothermal Method	[103]
Bamboo	*Bambusoideae*	N,S-CDs demonstrated remarkable stability, excitation wavelength-dependent emission, exceptional resistance to photobleaching, as well as tolerance to alkali and salt	Calcination Method	[104]
Devilwood	*Osmanthus*	Utilizing low-toxicity, high-concentration carbon-based nanomaterials for the selective creation of broad-spectrum antibacterial agents	Hydrothermal Method	[105]
Maple	*Acer*	Detection of cesium through biosensing and electrocatalytic glycerol oxidation	Hydrothermal Method	[106]
Mesquite	*Prosopis juliflora*	Intensely luminescent carbon dots and their utilizations in antibacterial and bioimaging applications	Hydrothermal Method	[107]
Vinca	*Catharanthus roseus*	Exploring dual fluorescence responsive behavior for multi-ion detection and biological applications	Hydrothermal Method	[108]
Carbon dots from fruits				
Lemon	*Citrus limon*	Optoelectronics and bioimaging, quenching features over bovine serum albumin (BSA) proteins, Mercury (II) Ion Sensing, and Live Cell Imaging	Hydrothermal Method, Microwave-Based Synthesis	[109–111]
Pomegranate	*Punica granatum*	Antimicrobial agent, prolific precursor for the production of carbon dots	Hydrothermal Method, Solvothermal Method	[112, 113]
Bael	*Aegle Marmelos*	Exploring Uncharted Territory: Crafting Carbon Dot Ratiometric Probes for Evaluating Solvent Polarity	Solvatochromic Method	[114]
Grapefruit	*Citrus paradisi*	Energy storage devices, Optical pH sensing, Packaging of meat products	Hydrothermal Method	[29, 115]
Mango	*Mangifera indica*	Tri-color Cellular Labeling (Blue, Green, and Red) and Metal Sensing on A549 Cells	Pyrolyzation Method	[116, 117]
Apple	*Malus domestica*	Imaging of mycobacterium and fungal cells	Hydrothermal Method	[20, 118, 119]
Zucchini	*Cucurbita pepo*	The utilization of ionic conductivity and diffusivity showcases the progression towards creating an eco-friendly supercapacitor product derived from agricultural residues	Hydrothermal Method	[120]
Papaya	*Carica Papaya*	Detecting Chromium in Water	Pyrolysis Method	[121]
Loquat	*Eriobotrya japonica*	Fluorescent Analysis of MnO4 − in Water Utilizing the Combined Influence of Inner Filter Effect and Static Quenching	Hydrothermal Method	[122]
Date palm	*Phoenix dactylifera*	Biomedicine plays a vital role in detecting biomolecules, transporting drugs, acting as fluorescent tracers, and regulating drug release	Microwave-assisted pyrolysis Method	[123]
Carbon dots from roots				
Ginseng	*Panax ginseng*	Kill cancer cells and inhibit malignant tumor	Solvothermal Method	[86, 124–126]
Beetroot	*Beta vulgaris*	High quantum yield and green CDs were obtained from Red Beetroot	Hydrothermal Method	[127, 128]
Radish	*Raphanus sativus*	Utilized as a sensing element in an optical electronic nose: Instantaneous detection of vaporized acetic acid	Hydrothermal Method	[129, 130]
Carbon dots from flowers				
Rose	*Rosa damascena*	An optimized, highly sensitive nanosensor utilizing the fluorescence quenching phenomenon of freshly synthesized carbon dots has been developed for the purpose of detecting Diazinon	Hydrothermal Method	[131]
Lily	*Lilium spp.*	Tetramethoxyporphyrin nanocomposite (CDs-TMPP)-based nano-effect NIR spectroscopy sensor	Hydrothermal Method	[132]
Orchid	*Orchidaceae*	Biocompatible fluorescent ink and also fluorescent probe for cellular imaging	Hydrothermal Method	[133]
Japanese camellia	*Camellia japonicah*	High near-infrared absorbance for efficient photothermal cancer therapy	Hydrothermal Method	[134]
Carbon dots from bark				
Cinnamon	*Cinnamomum verum*	Fluorescence Probes for Recognizing Cinnamaldehyde and l-Arginine/l-Lysine in Live Cells	Hydrothermal Method	[135]
Neem	*Azadirachta indica*	Effective inhibition of inflammatory MMP-9	Solvothermal Method	[136]
Carbon dots from husks				
Cocoa	Theobroma cacao	Bioimaging field, Drug delivery in the pharmaceutical industry	Microwave irradiation Method, Solvothermal Method	[137, 138]
Rice	*Oryza sativa*	Biocompatibility suggests biomedical applications,	Hydrothermal Method	[139–141]
Coconut	*Cocos nucifera*	Successfully converted into an innovative water disinfectant, Demonstrating efficient suppression of the self-fluorescence of papain enzymes	Hydrothermal Method, Pyrolysis, followed by sonication	[142, 143]
Carbon dots from rhizomes				
Turmeric	*Curcuma longa*	Determination of metanil yellow dye	Hydrothermal Method	[144]
Galangal	*Alpinia galanga*	Fluorescent sensor for Cd (II) determination	Hydrothermal Method	[145]
Mango Ginger	*Curcuma amada*	Sensing of hexavalent chromium	Microwave Method	[146]
Carbon dots from stems				
Aloe Vera	*Aloe barbadensis miller*	Polarity determines the interaction between precursors and the solvents and hence hydrophilic	Microwave Methods	[147]
Bamboo	*Bambusoideae*	Detection of Fe3 + Ions in Biological Systems	Hydrothermal Method	[148]
Eucalyptus	Eucalyptus spp.	Sensing the Synthetic Food Colorant and Bioimaging	Hydrothermal Method	[149, 150]
Carbon dots from seeds				
Chia	*Salvia hispanica*	Enhancing human safety while mitigating environmental pollution	Hydrothermal Method	[151]
Black cumin	*Nigella sativa*	Elective and quantitative detection of l-lysine (L-Lys) and tetracycline (TC)	Hydrothermal Method	[152]
Black sesame	*Sesamum indicum*	An Easily Accessible Method for Ammonia Vapor Detection	Hydrothermal Method	[153]
Cranberry Beans	*Phaseolus vulgaris*	Promising fluorescence probe for discriminative sensing of Fe3 + ions	Hydrothermal Method	[154]
Soya Bean	*Glycine max*	Detection of Fe3 + ions and cellular imaging	Pyrolysis Method	[155]
Lentil	*Lens culinaris*	Fe3 + ion detection	Hydrothermal Method	[156]
Groundnut	*Arachis hypogaea*	Sensor for Cr(VI) detection and in vitro bioimaging agent	Hydrothermal Method	[157]
Finger millet ragi	*Eleusine coracana*	Fluorescence probe for targeted Cu2 + detection	Pyrolysis Method	[158]
Carbon dots from peel				
Kiwi	*Actinidia Deliciosa*	The Fe3 + ion detection limit of K NCDs 1 and K NCDs 2	Hydrothermal Method	[159]
Orange	*Citrus sinensis*	Highly sensitive fluorescent switching sensor for Cr(VI) and ascorbic acid detection, and nano-booster for enhanced oxygen reduction reaction (ORR) electrocatalysis	Hydrothermal Method	[160, 161]
Garlic	*Allium sativum*	Photoluminescent carbon dots co-doped with nitrogen and sulfur for solar energy conversion, cellular labeling, and photobleaching applications	Pyrolysis Method	[162]
Banana	*Musa acuminata*	Visualization of human cancer cells through bioimaging	Hydrothermal Method	[163]
Apple	*Malus domestica*	Sunlight-induced photocatalytic degradation of crystal violet dye	Pyrolysis Method	[164]

### Extraction of natural products

Specific food items, like instant coffee and sugar beet molasses, may encompass natural carbon dots (NCDs) that can be directly extracted, obviating the requirement for synthesis. Efficaciously extracted NCDs from instant coffee through an uncomplicated procedure [18]. They introduced coffee powder into heated distilled water, subjecting the amalgam to vigorous stirring and subsequent centrifugation. The ensuing supernatant underwent filtration to eliminate sizeable particles, and the NCDs were subsequently refined through gel filtration chromatography. This method is straightforward and involves minimal steps. The NCDs obtained through this process exhibited excellent biocompatibility and were utilized for bioimaging in cells and fish. Similarly, Dinç extracted NCDs from sugar beet molasses without synthesis and employed them as biosensors for the analysis of riboflavin and tetracycline. However, it is important to note that this extraction method has limitations [19]. The extracted NCDs may have a wide size distribution, which can affect their properties and applications. Additionally, not all natural products contain NCDs, so this method cannot be universally applied. Nonetheless, the extraction of NCDs from food products provides a relatively simple and accessible approach for obtaining these nanomaterials with potential applications in various fields [20]. The effectiveness of synthesizing NCDs can be produced through a straightforward hydrothermal-carbonization method by utilizing *Chionanthus retusus* fruit extract as a carbon precursor and aqueous ammonia as a nitrogen source [21]. Intensely luminescent NCDs were generated utilizing an extract sourced from premature *Prunus mume* fruit, employing an uncomplicated single-step hydrothermal-carbonization method. The NCDs synthesis was executed across diverse pH spectra: 2.3, 5, 7, and 9. This was achieved by manipulating the pH of the Prunus mume extract through the introduction of a 25% aqueous ammonia solution [22]. Utilizing an age-old method akin to extracting fragrances from plant materials, red-emitting magnesium-nitrogen embedded carbon dots (r-Mg-NCD) were facilely fabricated from *Bougainvillea* leaf natural carbon extract. This extract was then carbonized through microwave treatment, yielding small-sized, photostable r-Mg-NCD exhibiting strong excitation-independent emissions at ~ 678 nm and a notable quantum yield of approximately 40% [23]. The C-dots produced at various temperatures were isolated and purified from the grilled hamburger samples. Comprehensive characterization encompassed their physical–chemical attributes, such as particle dimensions, fluorescence traits, lifetime, elemental composition, and surface functional groups [24]. Indeed, natural sources contain carbon dots (CDs). Certain researchers have managed to extract these CDs directly from natural materials using a straightforward approach involving centrifugation and filtration. This synthesis method eliminates the need for intricate procedures. Natural CDs were obtained by mixing coffee powder with hot water. These CDs exhibited a higher quantum yield (QY) of 5.5% compared to CDs derived from soy milk (2.6%). Cytotoxicity investigations have confirmed the safety of carbon dots (CDs) found in everyday food items, as they exhibited no harmful effects on cells even at concentrations as high as 20 mg mL^−1^. The researchers also successfully identified the presence of CDs in beer. Their technique encompassed a straightforward process involving condensation utilizing a rotary evaporator, succeeded by column chromatographic separation. Ultimately, CDs with an impressive quantum yield of 7.39% were obtained through lyophilization. Nevertheless, this extraction approach does come with certain limitations, notably the irregular sizes of the extracted CD particles and the inability to capture all-natural sources containing CDs [24].

### Hydrothermal carbonization

**Fig. 1:**
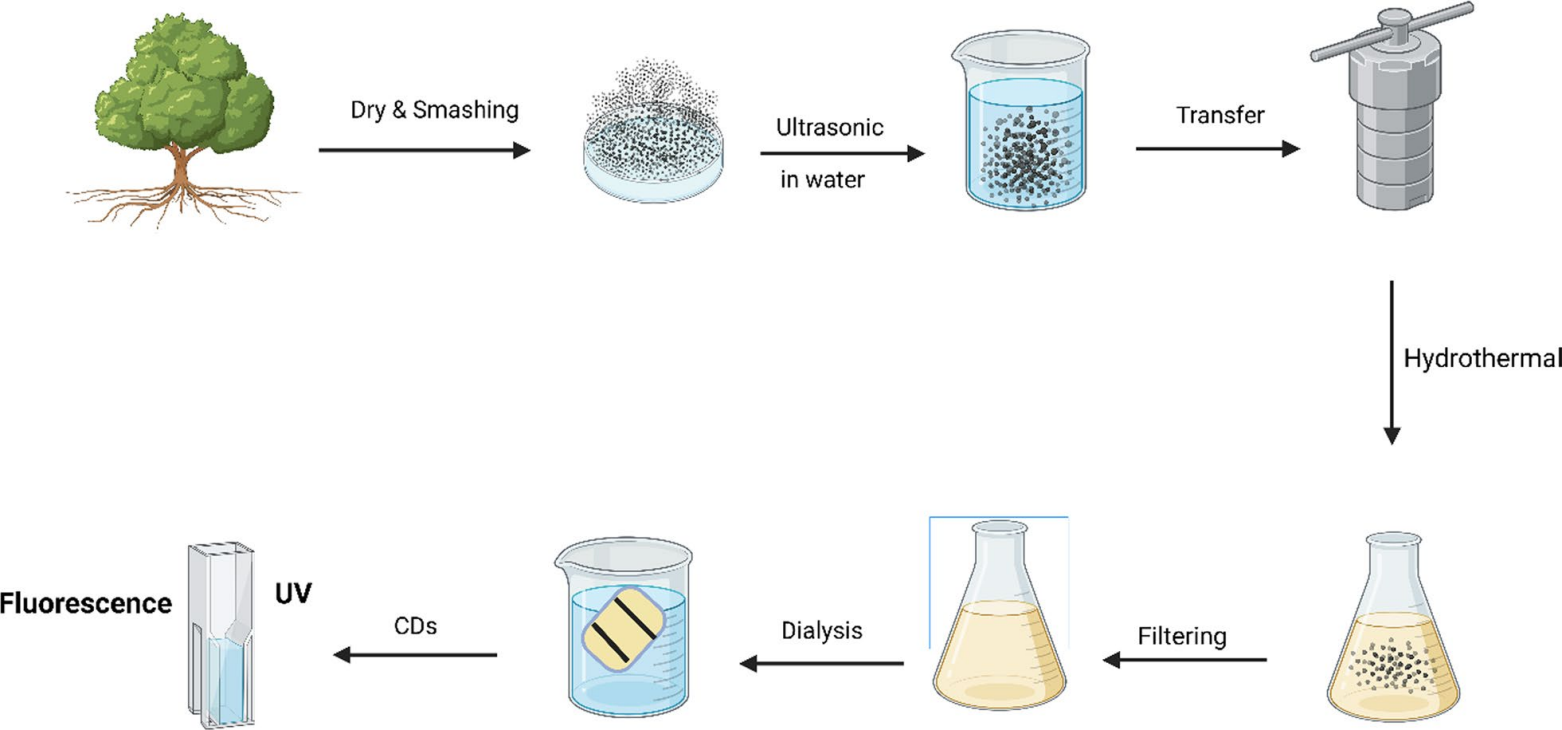
The process involves grinding dried herbs in pure water, treating with ultrasound, heating in a Teflon-lined autoclave, and then filtering and dialyzing for several days to achieve pure CDs. After that, there’s UV and Fluorescence

Hydrothermal carbonization stands as an economical, ecologically conscientious, and innocuous technique extensively utilized for the generation of pioneering carbon-centric substances originating from diverse natural carbon origins. These origins encompass apple juice, hemicellulose, bamboo leaves, cabbage, black tea, and a spectrum of biomass constituents [15, 25–29]. The process involves subjecting the carbon precursor to high temperatures within a sealed reactor. One notable application of hydrothermal carbonization is the production of nitrogen-doped carbon dots (NCDs) through the hydrothermal treatment of grass. This pioneering work was the first to report the successful preparation of NCDs with nitrogen doping using this method. Hydrothermal carbonization offers significant advantages as it enables the transformation of renewable resources into valuable carbon-based materials. Its simplicity, cost-effectiveness, and environmentally friendly nature make it an attractive option for the synthesis of novel carbon materials with various potential applications [30]. An effortless production of carbon dots (CDs) was achieved by employing used coffee grounds as the carbon reservoir, employing a hydrothermal approach. The CDs were integrated into a film composed of gelatin and poly (vinyl alcohol) (Gel/PVA), along with grapefruit seed extract (GSE), resulting in the creation of multipurpose packaging films. The multifaceted films' physiochemical attributes, functional characteristics, and their utilization in packaging were investigated [31]. Lemon peels (5 g) were cleaned, sun-dried, oven-dried (10 h, 100 °C), and crushed. Added to 100 mL 0.1 M H_2_SO_4_, then oven-dried (4 h, 100 °C) after water rinse and 5 min filtration. Treated with 150 mL sodium hypochlorite (4 h), pH-adjusted with water, hydrothermally reacted (12 h, 200 °C), dichloromethane-rinsed, centrifuged (10,000 rpm, 30 min) for water-soluble carbon quantum dots. Dried at 100 °C for final product [32]. Lemon peel waste was used to create water-soluble carbon quantum dots (wsCQDs) with a high quantum yield (QY) utilizing an easy and affordable hydrothermal procedure. With a quick response time, the hydrothermal synthesis technique has the benefits of green synthesis and resource conservation. The wsCQDs produced by this method have a limited size distribution and a nearly spherical shape, which suggests a constant and uniform particle size. With the help of this synthesis technique, it is possible to produce valuable carbon quantum dots from discarded lemon peel in a way that is both ecologically responsible and sustainable [33]. The hydrothermal process is the typical way to make carbon dots (CDs). Because it doesn't call for organic solvents or reagents, this method is beneficial for purification and subsequent usage in biological applications. Sadly, many CDs that have been reported have had low quantum yields in water, usually less than 5%. Their potential for other applications is constrained by this constraint. In 2016, Xiong et al. published a study introducing a hydrothermal approach for the one-pot synthesis of carbon dots (CDs) [11]. Moreover, in the pursuit of producing elemental-doped carbon dots (RCDs), scientists have explored carbon precursors incorporating supplementary elements. This approach offers benefits like regulated surface chemistry, controlled mesoscopic morphology, and tailored microstructures in the resulting functional CDs. To illustrate, carbonaceous materials for CD fabrication have been developed and employed alongside polythiophene derivatives. A technique for creating CDs with variable emission wavelengths between 482 and 680 nm under a single excitation wavelength of 400 nm was developed by Wang et al. The nitrogen (N) ingredient found in the CDs is principally responsible for the tunable emission in their work. These goods are ideal candidates for bioimaging applications because of their high biocompatibility [34]. However, in comparison to liquid biomass, the utilization of solid biomass may present a better solution for scaling up the synthesis process. This is primarily due to the longer shelf life of solid biomass and the convenience it offers in terms of scaling up the mass of precursors. By using solid biomass, it may be possible to achieve more efficient and practical large-scale production of carbon dots [35]. Using a hydrothermal process, carbon dots (CDs) co-doped with nitrogen and sulfur were made from garlic. The resultant CDs displayed favorable characteristics, such as excellent photo and pH stabilities, intense blue fluorescence emission, high fluorescent quantum yields of 17.5%, and acceptable water dispersibility. The CDs are suited for a variety of applications, including fluorescent labeling, bioimaging, and sensing, where stability, fluorescence intensity, and water compatibility are essential components [36]. A novel and environmentally friendly approach to synthesizing fluorescent nitrogen-doped carbon dots using milk as a starting material. The process is straightforward and efficient, offering a green alternative to conventional methods. By subjecting milk to hydrothermal heating, researchers successfully generated monodisperse carbon dots with a size of approximately 3 nm. These carbon dots exhibited high fluorescence properties. The study further demonstrated the suitability of these carbon dots as probes for imaging applications by successfully imaging U87 cells, a human brain glioma cancer cell line, with excellent resolution. This validation highlights the potential of the synthesized carbon dots for use in various imaging applications [37]. Hydrothermal synthesis stands out as environmentally friendly, excluding the use of organic matter. This is the primary rationale for producing natural-derived CDs. Additional passivation for the CDs' surface is unnecessary to ensure utmost safety and minimal toxicity. Before crafting, desiccated herbs are fragmented or ground into minute fragments within ultrapure water. The mixture is ultrasonically treated before being put into a Teflon-lined stainless steel autoclave and heated to a specific temperature. The combination must next be filtered via a 0.22 μm cellulose filtration membrane to ensure the purity of the carbon dots (CDs), and extensive dialysis over a period of days must follow in a dialysis pouch. The process of alteration is finished with these stages (Fig. 1).

### Pyrolysis method

**Fig. 2:**
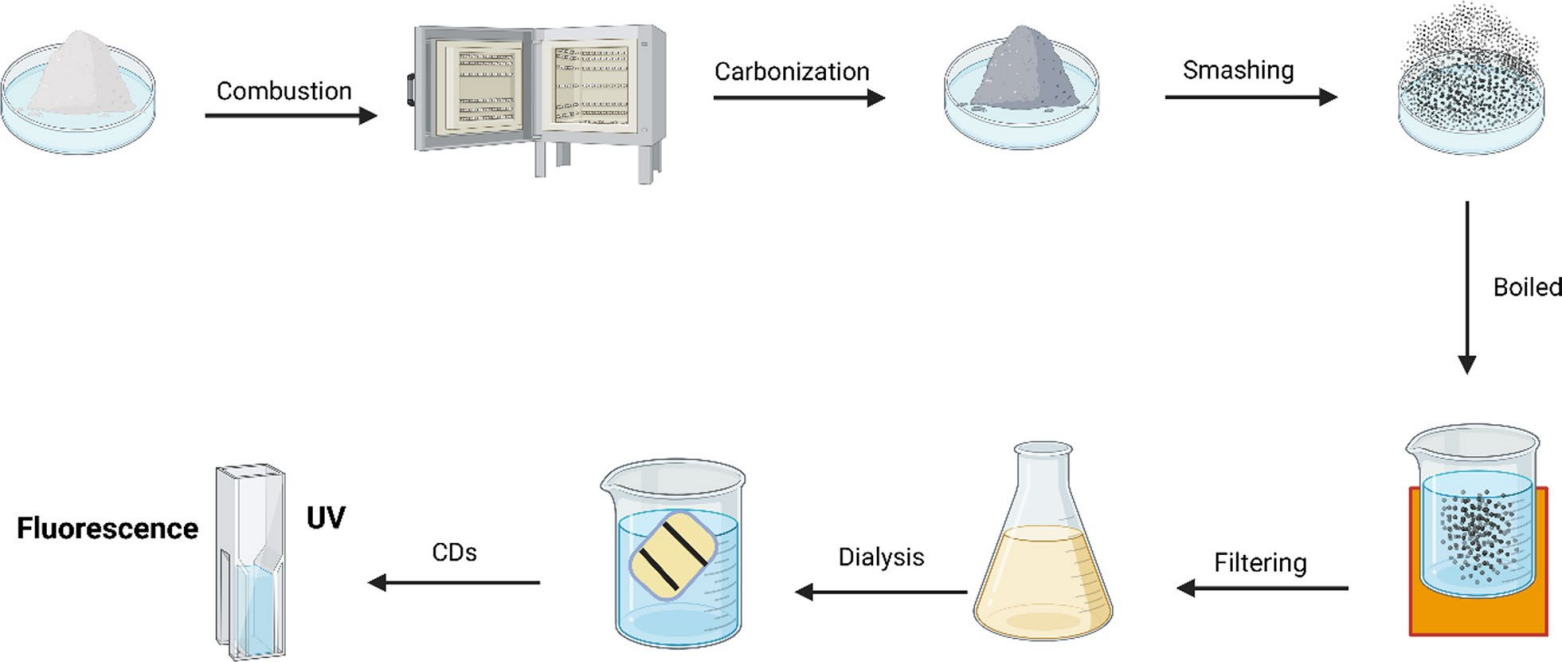
The process begins with herbal medicine placed in a crucible and heated precisely in a muffle furnace to induce carbonization. After pulverizing the resulting charred medicine and simmering it in ultrapure water, the upper liquid portion is collected. This solution is then filtered, ultimately yielding purified CDs. After that, there’s UV and Fluorescence

Pyrolysis stands as a method capable of transforming waste into nanomaterials. In this procedure, discarded tires undergo thermal decomposition, resulting in char, liquid, and syngas. The resulting pyrolytic char serves as a carbon-rich resource, containing carbon black and inorganic components used in tire production. The attributes of this char, such as surface area, chemical composition, and pore structure, can be improved via activation techniques [38]. Pyrolysis, a thermal decomposition process, is commonly employed to produce Biomass-Derived Carbon Dots (BCDs) [39]. These BCDs are typically synthesized with biomass serving as the carbon source. The particle size of BCDs prepared through pyrolysis typically ranges from 0.4 to 6 nm, while the quantum yield falls between 3 and 25%. Various plant materials, including rice husks [31], plant leaves [40], grass [41], and gynostemma [42], have been utilized for BCD synthesis via pyrolysis. For instance, BCDs have been successfully prepared through pyrolysis using rice husk biomass as the carbon source [31]. Solid-phase pyrolysis emerges as a highly efficient method for generating nanoparticles on a substantial scale, obviating the requirement for solvents. Additionally, the direct interaction among the reactants accelerates the reaction kinetics, leading to reduced energy expenditure throughout the synthesis process. This underscores the economic viability and enhanced efficacy of solid-phase pyrolysis for crafting CDs [43]. It is worth noting that the pyrolysis method can be applied to various biomass sources such as eggs and hair [44, 45]. For the first time, a cost-effective and naturally sourced material, konjac flour, has been harnessed as a carbon precursor to synthesize NCDs via a single-step pyrolysis approach. This innovative process yields NCDs with a remarkable quantum yield (QY) of 22%. These newly formed NCDs exhibit robust, vibrant photoluminescence (PL) characterized by a diverse range of colors. Notably, the intensity of this photoluminescence can be finely adjusted by employing distinct molecular weight variants of PEG for surface passivation or by introducing acids, alkaline substances, and amino acids [42]. A part from plant sources, waste materials can also serve as carbon sources for BCD synthesis via pyrolysis. Examples of such waste materials include litchi seeds [44], litchi exocarp [45], coffee grounds [46], waste frying oil [47], watermelon peel [48], peanut shell [49], peanut skin [43], and similar substances. A straightforward pyrolysis method can be employed to prepare water-soluble fluorescent BCDs using litchi seeds as the carbon source [44]. Despite the availability of alternative methods, some researchers still favor the pyrolysis approach for synthesizing carbon dots (CDs) from precursor molecules. Pyrolysis, being a thermal deposition method, is characterized by its irreversible nature. It involves subjecting various samples or organic materials to decomposition under inert conditions. This process induces physical and chemical changes in the organic samples, resulting in the formation of carbon-containing solid residues. To achieve this, controlled pressure and extremely high temperatures are employed during the pyrolysis process [50]. A groundbreaking methodology has been unveiled, encompassing the pyrolysis of trisodium citrate followed by ultrafiltration, as a means to craft monolayer graphene quantum dots (GQDs) characterized by exceedingly minute lateral dimensions measuring 1.3 ± 0.5 nm. The synthesis and subsequent utilization of these ultra-small GQDs, with their precisely controlled sizes, carry substantial import, particularly within the realm of biological applications [51]. By means of solid-phase pyrolysis, scientists effectively generated blue- and green-emitting fluorescent carbon dots, designated as GCDs-b and GCDs-g, respectively, featuring glycine enrichment. These carbon dots exhibited impressive quantum yields of 84.0% and 63.0%, along with significant product yields of 71.5% and 64.5%, respectively. These particles manifested uniform, spherical shapes, with average sizes measuring 4.1 nm for GCDs-b and 3.5 nm for GCDs-g. Furthermore, researchers conducted a comprehensive cellular viability assessment on GCDs-b and GCDs-g, affirming their excellent biocompatibility. This robust compatibility ensures the potential applicability of these materials in the realm of cellular imaging [52]. The herbal medicine is first placed into a crucible and exposed to precise heating using a muffle furnace, leading to carbonization. The resulting charred herbal material is then finely ground and simmered in ultrapure water, after which the upper liquid portion is collected. This solution is subsequently filtered through a 0.22 μm microporous membrane, followed by dialysis within a dialysis bag for several days, enabling the isolation of purified carbon dots (CDs) (Fig. 2).

### Solvothermal methods

Solvothermal synthesis is a successful method for the synthesis of chemical compounds, much like the hydrothermal process. The main distinction in solvothermal synthesis is the use of a non-aqueous precursor solution. With this technique, it is possible to precisely regulate the size, distribution of shapes, and crystallinity of nanostructures. The ensuing nanoarchitectures can be subject to modification by manipulating various experimental variables, encompassing reaction temperature, reaction duration, solvent composition, surfactant nature, and precursor variant. Solvothermal synthesis manifests as an adaptable avenue for nanomaterial production, as it permits the tailoring of material traits [53]. Solvothermal reactions are a specific type of synthetic method that utilizes organic solvents, such as benzene, DMF (dimethylformamide), or DMSO (dimethyl sulfoxide), instead of water to achieve desired reaction products. The choice of organic solvents plays a crucial role in determining the size and shape of the resulting products. Therefore, it is essential to thoroughly examine the chemical nature and physicochemical properties of these solvents to ensure successful outcomes [54].

### Microwave methods

Microwave chemistry is a very effective and quick way to make carbon dots (CDs). Benefits accrue from features like in situ and evanescent heating, which have the potential to significantly enhance both yield and quality of ultimate outcomes. A prevalent procedure entails solubilizing the natural substrate within a solvent, then subjecting the solution to microwave irradiation within a designated chamber. The resultant nitrogen-doped carbon dots (NCDs) can subsequently undergo segregation and refinement. In a work by Wang et al., they used wool as the starting material and a one-step microwave-assisted pyrolysis process to create NCDs. Unusually, this technique didn’t need for the use of any extra chemicals, underscoring its effectiveness and simplicity [55]. Utilizing a microwave-assisted process, nitrogen-doped carbon dots (NCDs) have been successfully synthesized. This innovative approach operates at a notably low temperature of 160 °C, yielding polymer nanodots derived from small organic molecules. These nanodots exhibit a photoluminescence center in a molecular state, resulting in an exceptional quantum yield of approximately 51.61%. The utility of NCDs extends to various applications, including the detection of metal ions, as well as fluorescence imaging of animal and plant cells. These applications are facilitated by the outstanding water solubility and minimal cytotoxicity exhibited by the NCDs, further enhancing their attractiveness in scientific and biomedical contexts [56]. The nitrogen-doped carbon dots (NCDs) endowed with a profusion of heteroatoms demonstrated a conspicuous photoluminescence efficiency of 17.1%. Furthermore, the utilization of flour as a carbon reservoir in the microwave-driven NCD synthesis underscores the multifaceted nature of microwave chemistry in harnessing a spectrum of carbon origins for NCD formulation [57]. To address the issue of long reaction times observed in previous preparations of nitrogen-doped carbon dots (NCDs) from natural products, flour was selected as the raw material. Compared to earlier methods, where reaction times could extend up to 3 h, the use of flour as a carbon source significantly reduced the synthesis time. In just 10 min of microwave heating, the NCDs were successfully obtained, offering a substantial improvement in reaction efficiency and time-saving benefits [58]. In the context of the synthesis process involving nitrogen and sulfur doped carbon dots (N, S-CDs), achieved through a microwave-assisted reaction between glycerol and cysteine, a notable application has emerged. This application pertains to the detection of Hg2 + cations [59]. The nitrogen-doped carbon dots (NCDs) prepared in this manner displayed a notable quantum yield of 16.3%. These NCDs were subsequently employed alongside silver nanoparticles to detect the herbicide glyphosate. Feathers, prevalent byproducts of the poultry sector, predominantly consist of β-keratin and encompass substantial proportions of carbon, nitrogen, sulfur, and oxygen. Acknowledging the feathers’ prospective value, Liu et al. devised a streamlined and effective route for crafting nitrogen and sulfur doped carbon dots (N, S-CDs) using goose feathers. This synthesis method involved a microwave-induced hydrothermal reaction, utilizing a 2 kW microwave power source [60]. Carbon dots, a burgeoning subset of carbon structures, have garnered substantial interest for their versatile and adjustable physico-chemical and optical traits. These nanomaterials, sized below 10 nm, offer a diverse range of properties via various synthesis routes, applicable across fields like sensing, bioimaging, solar cells, and catalysis. This review delves into their synthesis, particularly microwave-assisted approaches, and explores their multifaceted applications while addressing associated challenges and prospects [59].

### Chemical oxidation

Chemical oxidation presents a novel avenue for CD synthesis, typically involving potent oxidants such as sulfuric acid or nitric acid to initiate precursor oxidation and exfoliation. CDs produced via this technique often exhibit a profusion of functional groups, rendering them highly versatile for sensor applications and beyond [61]. When creating nitrogen-doped carbon dots (NCDs), natural materials can be carbonized with the use of chemical oxidants like hydrogen peroxide and oxidizing acids. Usually, the natural products are treated with a chemical oxidant first, then they are carbonized. After separation and purification, the resultant NCDs can be used. In a research published in 2012, Suryawanshi et al. described how dried neem leaves were oxidized with a solution of sulfuric and nitric acids to produce NCDs [18]. A pioneering top-down methodology was devised, combining chemical oxidation with a straightforward exfoliation procedure, to environmentally synthesize fluorescent CDs from discarded sugarcane bagasse pulp. The resulting CDs showcased notable attributes, including a substantial fluorescent quantum yield (approximately 18.7%), impressive crystalline structure, and remarkable biocompatibility. Notably, sugarcane bagasse itself was harnessed as the carbon source, further exemplifying the sustainable nature of this approach [62]. It also included instructions on how to oxidize colorful carbon dots. In a different work, Yan et al. created NCDs by chemically oxidizing starch, which were then used in a new approach based on fluorescence quenching to identify the chemotherapeutic medication imatinib [63]. Chemical oxidation is a practical way to alter the surface characteristics and fluorescence emission of nitrogen-doped carbon dots (NCDs), but it has certain drawbacks of its own. The probable existence of oxidation reagent residues in the produced NCDs is a serious disadvantage. The major goal of using NCDs for their biocompatible qualities is at odds with the residues' potential to contribute to increased biological toxicity. To guarantee the safe and successful use of NCDs in a variety of applications, it is crucial to take these problems into serious consideration and solve them [55].

## Physical and optical properties for natural products derived carbon dots

**Fig. 3:**
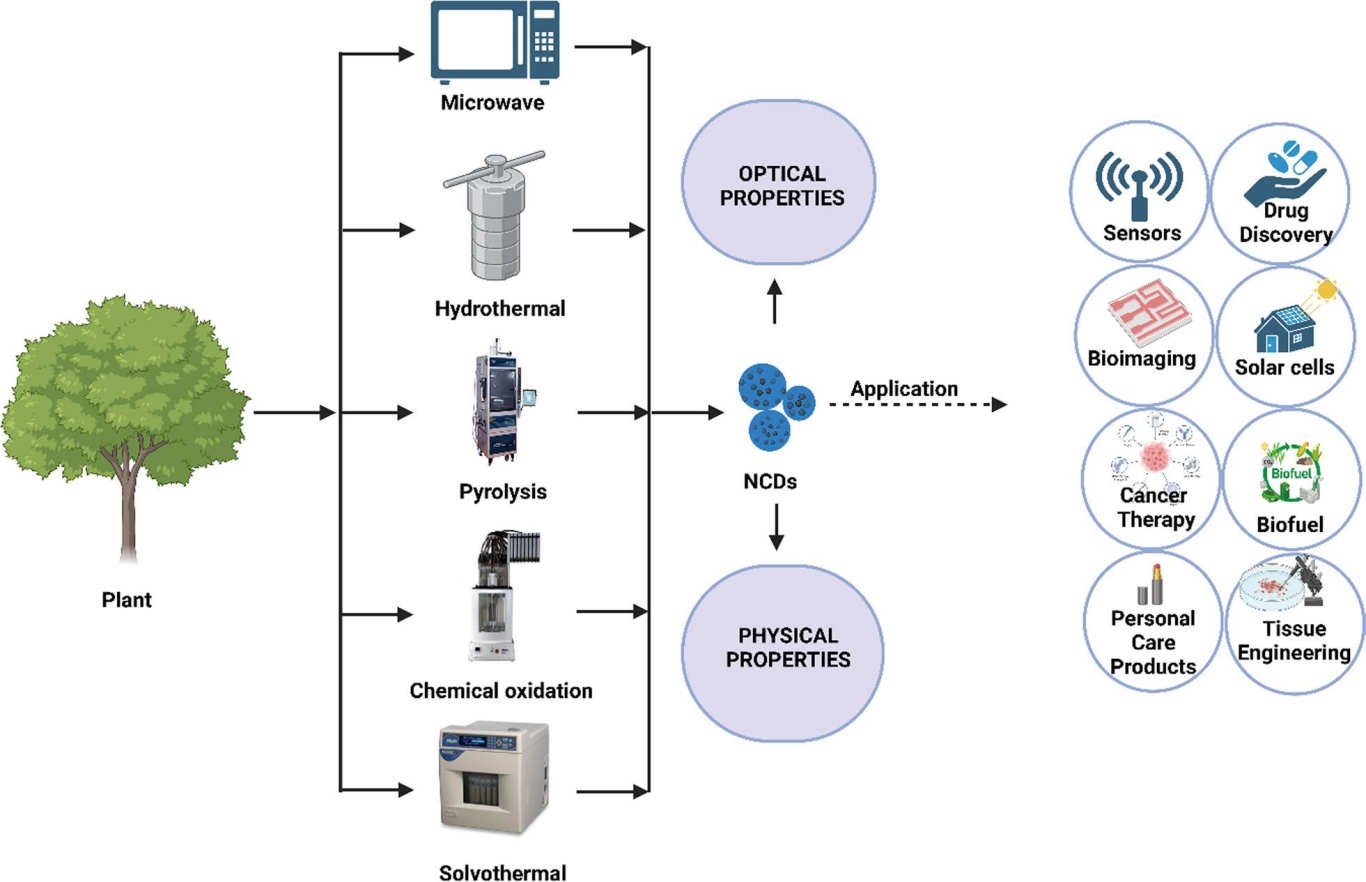
Plant parts are synthesized by various methods like hydrothermal, pyrolysis, chemical oxidation, solvothermal, and microwave to obtain naturally derived carbon dots. Carbon dots display unique physical and optical properties that make them extremely valuable for a wide range of applications, including bioimaging, tissue engineering, personal care products, solar cells, sensors, and drug discovery

NCDs exhibit distinctive physical and optical characteristics that render them exceptionally valuable across diverse applications (Fig. 3). Their distinctive physical and electronic traits hold particular promise in the realms of catalysis and energy storage implementations. The tunable electronic structure of NCDs allows for efficient electron transfer processes, making them suitable as catalysts or electrode materials in energy storage devices such as batteries and supercapacitors. Additionally, the high surface area and controllable surface chemistry of NCDs contribute to their exceptional catalytic activity and stability [64–66].

### Physical properties

#### Components and structure of NCDs

Natural carbon dots (NCDs) conventionally encompass carbon and oxygen components; however, when crafted from heteroatom-rich natural sources like hair and protein, they can additionally incorporate nitrogen and sulfur. N,S-CDs, showcase unparalleled optical and electronic attributes arising from the conjugation between the lone-pair electrons inherent in heteroatoms and the p orbitals associated with carbon atoms. Structurally, natural carbon dots generally exhibit a blend of amorphous and crystalline domains, interspersed with surface functional clusters. While certain scholars have observed crystalline segments containing sp2 carbon within NCDs, the overarching crystallinity in most instances remains relatively modest. Furthermore, NCDs often bear intricate surface groups, particularly those tethered to oxygen functionalities [60, 66–68].

#### Photoinduced electron transfer

Currently, there is significant ongoing investigation into the photoluminescence (PL) mechanism of carbon dots (CDs). Numerous hypotheses have been put forward, encompassing concepts such as PL arising from quantum confinement akin to conventional quantum dots, the creation of fluorophores during synthesis, and the involvement of energy traps in surface states. Evidently, additional research is imperative to ascertain a conclusive comprehension of the precise mechanism underlying the photoluminescence exhibited by carbon dots [69]. NCDs have the ability to act as both electron acceptors and donors. Nevertheless, the incorporation of electron acceptors may potentially diminish this fluorescence due to the occurrence of photoinduced electron transfer between the nitrogen-doped carbon dots (NCDs) and the electron acceptor. Conversely, NCDs frequently manifest fluorescence emission. However, they can also serve as formidable electron acceptors, thereby effectively dampening the luminescence emanating from recognized electron donors such as N, N-diethylaniline. This behavior demonstrates how NCDs are capable of participating in electron transfer processes and have a variety of electrical characteristics [70]. These investigations have primarily centered around the electron transfer to or from particular organic molecules, whether they are freely present, covalently linked, or electrostatically associated. However, the changes that occur in functional groups as a result of electron transfer to or from CDs remain ambiguous. Furthermore, the interplay between carbon dots and metal cations has also been a topic of discussion, giving rise to diverse theories such as the formation of charge transfer complexes [69, 71, 72]. Solvent affects the previously noted behavior of photoinduced electron transfer in NCDs, with increased efficiency shown in polar solvents. In both NCD-based photocatalysts and NCD-sensitized materials, this phenomenon is essential. These materials can display improved photochemical reactions and photocatalytic characteristics by taking use of NCDs’ capacity to promote electron transport. The total performance of materials made from NCDs in diverse applications is affected by the solvent environment, the interactions of the NCDs with the surrounding molecules, and these electron transfer mechanisms [64]. Indeed, when it comes to photoinduced electron transport, regular carbon dots (CDs) also show comparable characteristics. Wang et al. also noted comparable outcomes using regular CDs. The surface moieties on the NCDs, the production and dynamics of electron–hole pairs, and the ensuing recombination processes all have an impact on the photoinduced electron transfer behavior in the case of NCDs. These variables work together to affect the optical and electrical properties of NCDs by determining the efficiency and features of photoinduced electron transfer [73].

### Optical properties

The fluorescence emission and optical absorption within carbon dots (CDs) arose from the mechanistic interplay of plasmon-induced phenomena and radiative recombination involving surface-bound electrons and holes, as opposed to a conventional band gap. This mechanism diverges from the process observed in conventional semiconductor quantum dots [28].

#### Aggregation-Induced-Emission (AIE) of CDs

Though the majority of carbon dots (CDs) have demonstrated remarkable photoluminescence (PL) in solution or dispersed states, instances of CDs exhibiting aggregated or solid-state PL have been relatively scarce. However, recent strides have been made in the domain of Aggregation-Induced Emission (AIE) CDs, wherein these CDs congregate into nanostructures due to restricted intermolecular motion. The AIE characteristics of these CDs permit the creation of proficient photosensitizing or photothermal agents for Photodynamic Therapy (PDT) or Photothermal Therapy (PTT), as well as enabling compact packing within LEDs while upholding PL efficiency. In a recent investigation conducted by Hu et al., hydrophobic CDs (HCDs) were effectively synthesized through a one-step hydrothermal process, involving melamine (MR) and dithiosalicylic acid/acetic acid [74].

#### Absorbance

With a modest expansion into the visible range, NCDs (Nitrogen-doped carbon dots) typically display optical absorption that is concentrated in the ultraviolet (UV) region of the electromagnetic spectrum. The absorption behavior in NCDs arises from several factors, including the n–p* transition of C=O bonds, the p–p* transition of C–C bonds, and potentially other transitions. The limited capacity to develop extensive conjugated graphene domains within NCDs due to the methods of synthesis employed may fundamentally contribute to the confined absorption spectrum observed in them [66, 70]. Lin et al. reported the synthesis of mCDs (Multimode-emissive CDs) using polyvinyl alcohol (PVA) and phenylenediamine (PD). The ethanolic solution of these mCDs exhibited two clearly distinguishable absorption bands. The first absorption band was observed at 247 nm, which can be attributed to the π–π* transition of the C=C bonds present in the structure of the CDs. The second absorption band appeared at 355 nm and corresponds to the n-π* transition of the C–N/C=N bonds in the CDs [75]. In a work by Nie et al., l-cysteine and d-cysteine were used to create extremely luminous chiral CDs. The L-CDs’ UV spectra showed two distinct absorption bands. The aromatic sp2 transition of the CDs’ initial absorption band, which emerged at 243 nm, was attributed to it. The n-* transition of C=O/C–N/C–S bonds was detected in the second absorption band at 300 nm. At 400 nm, a weaker absorption band was further seen. d-cysteine, on the other hand, showed no sign of an absorption band over 240 nm [76].

#### Fluorescence in UV–Vis and NIR regions

The exploration of NCD quenching encompassed an inquiry into the interplay between NCDs and an array of nitroaromatic compounds featuring distinct ring substituents. Based on empirical observations, it has been determined that the primary mechanism responsible for quenching nitrogen-doped carbon dots (NCDs) is Forster resonance energy transfer (FRET), rather than electron transfer. However, the fluorescence emission mechanism of NCDs has received less attention. Given the similarity in fluorescence behavior between CDs and NCDs, it is plausible to consider some of the mechanisms proposed for CDs to explain the fluorescence emission of NCDs. These mechanisms encompass surface passivation/defects [77], quantum size effect [78], carbon-Core state effect, surface state effect, and edge state. These mechanisms may contribute to the fluorescence emission of NCDs, but further research is needed to establish their specific roles and contributions [79]. In general, CDs exhibit fluorescence characteristics that depend on the excitation wavelength. However, there have been reports of excitation-independent emission in N, S co-doped CDs. Pang et al. utilized a precisely controlled wet oxidative method to synthesize excitation-independent CDs with tunable fluorescent colors. Their findings indicated that the photoluminescence (PL) properties of CDs were primarily influenced by their molecular weight and the extent of surface oxidation [80, 81].

## Applications of natural products derived carbon dots

Green CDs offer distinct advantages over chemically derived CDs due to their unique properties. These include reduced toxicity, excellent water dispersibility, superior biocompatibility, remarkable photostability, vibrant fluorescence, and convenient modification capabilities. As a result, these nanomaterials hold great promise in the fields of sensor technology and biology. They particularly excel in electrochemical sensing of toxic and trace elements in ecosystems, metal detection, disease diagnosis through bio-sensing, as well as in-vitro and in-vivo bio-imaging applications for detecting cancerous cells [82]. In recent years, scientists have increasingly utilized green-synthesized CDs to achieve selective sensing of various biomolecules, including carbohydrates, amino acids, glutathione, and hydrogen peroxide (Fig. 3). The biosensing mechanisms employed in these studies are comparable to those used in chemical sensing techniques. For instance, CDs derived from date kernels were successfully employed in the determination of zoledronic acid levels in human serum. Another example involves the synthesis of NCDs from Konjac flour using pyrolysis treatment, which enabled the development of an off–on sensing system for detecting L-lysine. These examples highlight the diverse applications and potential of green CDs in the field of selective biomolecule sensing [42, 83]. However, it is worth noting that CDs derived from citric acid exhibited significant cytotoxicity at a concentration of 400 μg/mL. Conversely, Sahu and colleagues achieved the successful creation of non-toxic carbon dots (CDs) boasting a fluorescence quantum yield of 26%. This feat was accomplished through the utilization of a hydrothermal treatment technique, employing orange juice as an easily accessible natural bioresource. This groundbreaking study represents the first example of fluorescent NCDs being synthesized from such easily accessible natural sources [84, 85]. Natural product-derived carbon dots (NPdCDs) have emerged as promising tools in various fields, including cancer research, microbial imaging, drug sensing, and drug delivery. These carbon dots, derived from bioactive phytomolecules found in plants, possess remarkable medicinal properties, biocompatibility, photo-stability, and easy functionalization. Their versatility has led to widespread applications across diverse domains. In particular, NPdCDs have shown significant advancements in the field of cancer biology. The purpose of this review is to discuss recent breakthroughs in the utilization of NPdCDs, focusing specifically on their role in cancer research [86]. Pesticides, which are chemical-based substances, are employed to protect crops from various threats such as rodents, weeds, fungi, insects, and pests. However, it is important to note that pesticides can be toxic to humans. They are classified into different categories based on their specific target, such as herbicides for weeds, fungicides for fungi, and insecticides for insects. Within the realm of pesticides, carbamate pesticides and organophosphorus compounds have been widely utilized in agriculture due to their broad spectrum of activity and high effectiveness. Deka et al. [87]. Functional CDs have demonstrated their significant potential as catalysts for the degradation of dye contaminants in water applications. One of the key reasons behind their catalytic capability is their ability to act as electron mediators, serving as both donors and acceptors. This unique characteristic makes them promising catalysts in this context. Compared to other photocatalysts like TiO_2_, ZnO, and CdS, the synthesized green CDs exhibit satisfactory photocatalytic activity. This is attributed to their higher chemical stability, superior water solubility, and lower toxicity. Moreover, green CDs possess light absorption properties in the near-infrared (NIR) region. This ability to absorb NIR light enables them to function effectively as catalysts, as high-energy radiation in this range can break down organic compounds [88]. In a research endeavor led by Huang and co-workers, nitrogen-doped carbon dots (NCDs) were synthesized via a microwave-based methodology. These NCDs were deliberately engineered with a focus on detecting a specific sequence linked to the colitoxin gene. The developed NCD-based system has potential applications in determining colitoxin DNA in human serum, offering a promising approach for the diagnosis and monitoring of colitoxin-related conditions or diseases [89]. In their work, Zhang et al. introduced a fluorescent quenching principle that significantly enhances the conversion efficiency of NCD-sensitized aqueous solar cells [90]. Carbon dots derived from diverse biomass sources like chitin, chitosan, and glucose were employed in the construction of solid-state solar cells, yielding varying levels of performance. These disparities in efficacy were attributed to the distinct functional groups present on the surface of the nitrogen-doped carbon dots (NCDs). In another study conducted by Choi et al., NCDs were prepared through the thermal decomposition of the oligosaccharide α-cyclodextrin. These NCDs were then utilized to support silver nanoparticles (NCD-Ag) for potential applications. This research demonstrates the versatility and potential of NCDs in various fields, including solar cell technology and nanomaterial synthesis [91].

## Challenges of natural product carbon dots

Carbon dots (CDs) are gaining prominence as a versatile solution for a wide array of environmental and energy applications. Their extraordinary physicochemical, optical, and electrical attributes render them ideal for a multitude of applications, spanning chemical catalysis, photocatalysis, electrocatalysis, and energy storage implementations such as batteries and capacitors [165]. Nevertheless, despite the promising nature of multifunctional carbon dots synthesized from natural sources, challenges persist in this exciting field. A recent review, published in Environmental Science and Pollution Research, delves into the latest advancements in carbon dots, focusing on their biological and environmental applications. The review provides valuable insights into the current progress, highlighting the potential of these carbon dots in environmental and energy-related fields, while also addressing the existing challenges and promising prospects in this area [166]. Carbon dots (CDs) possess distinctive attributes that render them highly valuable in a wide range of environmental and energy applications. Despite this, achieving CDs with desired features, such as broad photoluminescence and controllable surface groups, remains challenging [167]. Recently, researchers have shown significant interest in CDs as a potential alternative to toxic metal-based quantum dots (QDs). These novel carbon nanomaterials exhibit a small size with a core–shell-like structure and offer tunable photoluminescence (PL), akin to quantum dots (QDs). Notably, carbon dot fluorescence involves absorbing high-energy photons and emitting lower energy ones, while certain C-dots can undergo multiphoton excitation, enabling them to convert longer wavelengths of light into higher energy photons [165, 168, 169]. CDs, as nanoparticles, exhibit versatile physicochemical and optical characteristics that can be adjusted according to specific needs. Their notable resistance to photobleaching and relatively low toxicity make them highly appealing as substitutes for fluorescent dyes and quantum dots based on heavy metals. As a result, CDs find applications in various fields, including bioimaging, sensing, catalysis, solar cells, and light-emitting diodes. Their adaptability and safer profile contribute to their growing popularity in these diverse areas of research and application [169]. Additionally, the review also addresses the potential future opportunities and challenges in utilizing CDs for resolving environmental issues. The exploration of these aspects provides valuable insights into how carbon dots can further contribute to tackling environmental challenges, paving the way for sustainable and innovative solutions in the future [170]. However, biosensing encounters certain challenges, primarily arising from the susceptibility of their bioreceptors. For instance, deoxyribonucleic acid (DNA) is prone to degradation, necessitating specific storage and analysis conditions. Similarly, the instability of enzymes can lead to sensor ineffectiveness, especially in high-temperature environments. Addressing these vulnerabilities is crucial to enhance the reliability and efficiency of biosensors for various applications [171]. Moreover, the integration of nanomaterials into biosensors has the potential to significantly enhance the properties of bioreceptors, thereby expanding their application range. An array of nanomaterials, encompassing metal nanoparticles, quantum dots based on semiconductors, materials infused with dyes, carbon-based nanomaterials, MOFs (Metal organic frameworks), and COFs (Covalent organic frameworks), have been harnessed for the purpose of sensing and investigating biomolecules. Nanomaterials with exceptional optical and electrochemical characteristics have proven highly successful and garnered significant attention in biosensing research. However, despite their advantages, nanomaterials-based biosensors also encounter certain challenges that require attention and further investigation for continued improvement and widespread implementation [172]. Carbon dots (CDs) possess exceptional properties that make them highly versatile for a wide range of environmental and energy applications. Nonetheless, achieving C-dots with superior performance, such as broad photoluminescence and controllable surface groups, remains a challenging task [173]. Recently, CDs have garnered significant interest among researchers as a potential alternative to toxic metal-based quantum dots (QDs). These novel carbon nanomaterials exhibit a compact size with a core–shell-like structure and demonstrate tunable photoluminescence (PL) akin to quantum dots (QDs). In carbon dot fluorescence, high-energy photons are absorbed, leading to the emission of lower energy photons. Some specific carbon dots can even undergo multiphoton excitation, allowing them to convert longer wavelengths of light into higher energy photons [174]. However, one of the hurdles in working with carbon dots is their heterogeneity, which presents challenges in achieving consistent and controlled properties across different batches of these nanomaterials. Overcoming this issue is crucial to unlocking their full potential in various applications and maximizing their benefits in environmental and energy-related fields [175, 176]. The sensing methods of these biosensors will be thoroughly examined and analyzed, along with a comprehensive discussion of their respective advantages and disadvantages. The application scopes of these biosensors in different fields will also be carefully assessed and explored [172].

## Future prospects of natural product carbon dots

CDs show potential in various biomedical applications, including bioimaging, drug delivery, and theragnostic (combined diagnostic and therapeutic functions). Their biocompatibility and fluorescence properties make them excellent candidates for non-toxic bioimaging agents. They can also be functionalized to deliver drugs or act as carriers for targeted therapy [177–180]. CDs can be utilized as sensors for detecting environmental pollutants and monitoring water quality. Their fluorescence can be tuned to respond to specific environmental cues, making them valuable tools for pollution detection and environmental surveillance. Metal-Doped Carbon Dots as Resilient Nanomaterials for Monitoring and Degrading Water Pollutants [125, 181–183]. The unique electronic properties of CDs make them promising candidates for energy storage and conversion applications. They can be used as electrodes in supercapacitors and batteries, potentially enhancing energy storage capacity and performance [184, 185]. In contemporary times, their potential applications have extended across an array ranging from bio-imaging, fluorescent probing, and catalytic endeavors, to the realm of energy storage disciplines, notably as constituents within pivotal components of electrochemical energy storage mechanisms [186]. CDs have shown catalytic properties for various chemical reactions, such as the reduction of heavy metal ions or organic pollutants. They could potentially find applications in environmental remediation and green chemistry [187, 188]. By virtue of their modifiable fluorescence and distinct optical characteristics, carbon dots (CDs) hold the potential to contribute to optoelectronic apparatuses, including but not limited to light-emitting diodes (LEDs), lasers, and photodetectors [91, 189]. CDs derived from natural sources could be employed as additives in food packaging materials, helping to improve their barrier properties and enhance food preservation while reducing environmental impact [190–192]. CDs could be used in agricultural applications, such as nanofertilizers or nanopesticides, to improve crop yield and reduce the use of chemical inputs, thus promoting sustainable agriculture practices [193, 194]. Carbon dots possess unique quantum properties due to their small size and quantum confinement effects. They could potentially be used as quantum bits (qubits) in quantum computing systems, contributing to the development of more efficient and powerful quantum computers [195]. CDs could be engineered to target specific cancer cells or tumor tissues, offering a potential platform for targeted cancer therapy. They may also be used in photodynamic therapy, where they generate reactive oxygen species upon light exposure to kill cancer cells [196]. While carbon dots (CDs) have shown great promise for various applications, there are still limitations to address for their in vivo use. The exact mechanism of CDs is a subject of debate, partly due to variations in synthetic routes, precursor materials, surface functional groups, and size distribution. Designing CDs for in vivo applications requires considering factors like toxicity, blood compatibility, rapid clearance from the body, appropriate hydrodynamic diameter, and minimal protein adsorption. Overcoming these challenges will pave the way for safer and more effective applications of carbon dots in biomedical settings [197].

## Appropriate methods selection of natural product CDs production

Carbon dots (CDs), a recent addition to the well-known carbon-based nanomaterials category, have sparked significant interest across numerous domains [198]. Hydrothermal method is most widely used in the plants derived carbon dots. This approach, known for its eco-friendliness, non-toxic nature, affordability, and straightforward operation, found widespread adoption among researchers [199]. An issue encountered when using the hydrothermal method to synthesize plant-derived carbon dots is the limited yield and subpar product quality. This method entails high temperatures and pressures, leading to potential carbon dot degradation and aggregation. Additionally, the hydrothermal process necessitates extended reaction times and intricate purification procedures, ultimately diminishing the synthesis's efficiency and reproducibility [200, 201]. An alternative method for synthesizing plant-derived carbon dots is the microwave-assisted method. This method employs microwave radiation to swiftly heat and carbonize plant-derived materials while utilizing a compatible solvent and catalyst. The microwave-assisted process is capable of yielding a substantial amount of high-quality carbon dots with a narrow size range, intense fluorescence, and excellent stability in a relatively brief duration. Furthermore, this method stands out for its simplicity, convenience, and eco-friendliness, as it avoids the need for harsh conditions or toxic substances [202, 203].

When it comes to synthesizing plant-derived carbon dots using the pyrolysis method, a notable drawback is the limited crystallinity and subpar optical characteristics of the resulting products. This technique entails subjecting plant materials to high temperatures and rapid heating, leading to incomplete carbonization and the formation of irregular carbon dot structures. Additionally, the pyrolysis method demands exceptionally pure and uniform carbon sources, which can be challenging to procure from plant-based materials [43, 204]. Another viable approach to producing plant-derived carbon dots is through the hydrothermal method. This method utilizes water as both a solvent and a reaction medium to hydrolyze and carbonize the plant precursors under gentle conditions. The hydrothermal method has the capability to yield top-notch carbon dots characterized by high crystallinity, consistent size, intense fluorescence, and exceptional stability. Furthermore, this technique is known for its simplicity, convenience, and eco-friendliness, as it operates without the need for elevated temperatures or toxic chemicals [62, 205].

One of the problems for solvothermal method in synthesis of plant-derived carbon dots is the high cost and energy consumption of the process. The solvothermal method involves high temperature and pressure, which can cause safety hazards and equipment damage. Moreover, the solvothermal method requires organic solvents, which are expensive, toxic, and non-renewable [206, 207]. An alternative method for synthesizing plant-derived carbon dots is the green synthesis method. This method uses natural and renewable plant materials as carbon sources and water or mild acids as solvents to produce carbon dots under mild conditions. The green synthesis method can produce high-quality carbon dots with low cost, low toxicity, and high environmental friendliness [169, 208].

Microwave method in synthesis of plant-derived carbon dots is the low reproducibility and stability of the products. The microwave method involves rapid and uneven heating, which can cause variation in the size, shape, and surface chemistry of the carbon dots. Moreover, the microwave method requires precise control of the reaction parameters, such as power, time, temperature, and pressure, which can affect the quality and yield of the carbon dots [59, 209]. An alternative method for synthesizing plant-derived carbon dots is the solvothermal method. This method uses organic solvents as both the carbon source and the reaction medium to produce carbon dots under high temperature and pressure. The solvothermal method can produce high-quality carbon dots with high reproducibility, stability, and fluorescence intensity. Moreover, the solvothermal method is flexible, scalable, and compatible with various plant precursors [210, 211].

The problems for chemical oxidation in synthesis of plant-derived carbon dots is the use of harsh and toxic chemicals, such as nitric acid, sulfuric acid, or potassium permanganate, which can cause environmental pollution and health hazards. Moreover, the chemical oxidation method requires high temperature and long reaction time, which can reduce the efficiency and yield of the synthesis [38, 178]. Alternative method for synthesizing plant-derived carbon dots is the green synthesis method. This method uses natural and renewable plant materials as carbon sources and water or mild acids as solvents to produce carbon dots under mild conditions. The green synthesis method can produce high-quality carbon dots with low cost, low toxicity, and high environmental friendliness [169].

## Conclusion

In conclusion, the recent surge in interest and research on natural products-derived carbon dots (NCDs) marks an exciting chapter in material science. These nanomaterials possess exceptional properties and offer numerous benefits, making them highly appealing for diverse applications across various fields. The utilization of abundant natural resources, such as leaves, fruits, roots, and more, highlights the eco-friendly nature of NCDs, presenting a sustainable alternative to conventional synthetic materials. As scientists and researchers continue to unravel their potential, the realization of sustainable, high-performance materials with minimal environmental impact seems within reach. The application of NCDs in various fields holds the promise of transforming industries and contributing to a greener and healthier future for humanity.

## Data Availability

The data related to manuscript will be provided on request.
